# Intimate partner violence, substance use, and health comorbidities among women: A narrative review

**DOI:** 10.3389/fpsyg.2022.1028375

**Published:** 2023-01-27

**Authors:** Jacqueline B. Mehr, Esther R. Bennett, Julianne L. Price, Nicola L. de Souza, Jennifer F. Buckman, Elisabeth A. Wilde, David F. Tate, Amy D. Marshall, Kristen Dams-O'Connor, Carrie Esopenko

**Affiliations:** ^1^School of Environmental and Biological Sciences, Rutgers University – New Brunswick, New Brunswick, NJ, United States; ^2^School of Social Work, Rutgers University - New Brunswick, New Brunswick, NJ, United States; ^3^Department of Kinesiology and Health, Rutgers University - New Brunswick, New Brunswick, NJ, United States; ^4^Center of Alcohol and Substance Use Studies, Graduate School of Applied and Professional Psychology, Rutgers University - New Brunswick, New Brunswick, NJ, United States; ^5^School of Graduate Studies, Biomedical Sciences, Rutgers Biomedical and Health Sciences, Newark, NJ, United States; ^6^Department of Rehabilitation and Movement Sciences, School of Health Professions, Rutgers Biomedical and Health Sciences, Newark, NJ, United States; ^7^Department of Rehabilitation and Human Performance, Icahn School of Medicine at Mount Sinai, New York, NY, United States; ^8^Department of Neurology, School of Medicine, The University of Utah, Salt Lake City, UT, United States; ^9^George E. Wahlen, VA Salt Lake City Healthcare System, Research Care Line, Salt Lake City, UT, United States; ^10^Department of Psychology, College of the Liberal Arts, The Pennsylvania State University, State College, PA, United States; ^11^Department of Neurology, Icahn School of Medicine at Mount Sinai, New York, NY, United States

**Keywords:** substance misuse, interpersonal violence, brain injury, resilience, mood disorders

## Abstract

Exposure to intimate partner violence (IPV), including physical, sexual, and psychological violence, aggression, and/or stalking, impacts overall health and can have lasting mental and physical health consequences. Substance misuse is common among individuals exposed to IPV, and IPV-exposed women (IPV-EW) are at-risk for transitioning from substance misuse to substance use disorder (SUD) and demonstrate greater SUD symptom severity; this too can have lasting mental and physical health consequences. Moreover, brain injury is highly prevalent in IPV-EW and is also associated with risk of substance misuse and SUD. Substance misuse, mental health diagnoses, and brain injury, which are highly comorbid, can increase risk of revictimization. Determining the interaction between these factors on the health outcomes and quality of life of IPV-EW remains a critical need. This narrative review uses a multidisciplinary perspective to foster further discussion and research in this area by examining how substance use patterns can cloud identification of and treatment for brain injury and IPV. We draw on past research and the knowledge of our multidisciplinary team of researchers to provide recommendations to facilitate access to resources and treatment strategies and highlight intervention strategies capable of addressing the varied and complex needs of IPV-EW.

## 1. Introduction

It is estimated that one in three women worldwide experiences physical and/or sexual violence from an intimate partner (Devries et al., 2013; Breiding et al., 2014; WHO, 2021). Intimate partner violence-exposed women (IPV-EW) frequently experience negative psychological (Campbell, 2002; Afifi et al., 2009; Fletcher, 2010; Okuda et al., 2011; Brignone et al., 2018; Iovine-Wong et al., 2019) and physiological (Campbell, 2002; Wuest et al., 2008, 2010; Kwako et al., 2011) symptoms, as well as comorbidities such as depression, anxiety, posttraumatic stress disorder (PTSD; Dardis et al., 2021), and suicidal ideation (Alhusen et al., 2015; Brignone et al., 2018; Iovine-Wong et al., 2019; Rasmussen et al., 2021). Substance misuse is also seen after IPV exposure in women (Testa et al., 2003; Pallatino et al., 2019; Ogden et al., 2022). Furthermore, the physical and sexual trauma that often occurs in IPV increases the risk for bodily injuries that can result in chronic pain, as well as head, neck, and/or facial injuries that can cause traumatic brain injury (TBI) or acquired hypoxic–ischemic brain injury as a result of non-fatal strangulation (NFS) or other forms of impeded breathing (Wilbur et al., 2001; Valera and Berenbaum, 2003; Wuest et al., 2008; St Ivany and Schminkey, 2016; Campbell et al., 2022; Walker et al., 2022)^1^. Large independent literatures have identified interrelationships between IPV exposure history and mental health diagnoses, chronic pain, brain injury, substance misuse, and substance use disorder (SUD; Merkel et al., 2017; Voon et al., 2018; Lutgendorf, 2019; Stone and Rothman, 2019; Campbell et al., 2022; Haag et al., 2022; Ogden et al., 2022; Oram et al., 2022); yet, few studies have attempted to piece together the complex interactions between these variables in a manner that supports frontline clinical strategies that more holistically bolster the quality of life for IPV-EW.

This narrative review begins this process by providing a broad framework for understanding substance use and misuse in IPV-EW to researchers and frontline clinicians. IPV and mental health comorbidities have been thoroughly discussed in the literature (Campbell, 2002; Afifi et al., 2009; Okuda et al., 2011; Brignone et al., 2018), and the varied and complex interplay of these factors on risk of substance misuse and SUD warrants a targeted discussion. Despite the elevated risk for substance misuse and SUD associated with IPV, the causal pathways from IPV to SUD and the interaction between IPV exposure and comorbid mental health diagnoses, pain, and brain injury is not clearly understood. This dearth of research exploring mediating factors in the relationship between IPV and SUD interferes with the development of new treatment targets and improvement of clinical outcomes for IPV-EW. Moreover, it perpetuates common stigmas associated with substance misuse, including the identification of substance users as intractable, “difficult” to treat, and even incurable.

## 2. The relationship of substance use and IPV exposure

Substance misuse is defined as substance use at high doses or in inappropriate situations. SUD is a diagnosable illness that arises following prolonged substance misuse and that significantly alters health and daily functioning (Campbell, 2002; McLellan, 2017). Substance misuse in IPV-EW has been reported as a means of coping with the physical and emotional pain (Smith et al., 2012; Simonelli et al., 2014; Gezinski et al., 2021). Self-medication, defined in the general population as using alcohol, recreational drugs with analgesic properties, and prescription opioids to treat pain (Alford et al., 2016; Cil et al., 2019; Rogozea et al., 2020), can accelerate the progression from substance use to SUD (Timko et al., 2005; Lehavot et al., 2014; Hogarth et al., 2019); however, the use of psychoactive substances as self-medication is not specific to the IPV community - it is a common pathway to addiction across populations. Moreover, similar to research in other fields that links heavy and frequent use of psychoactive substances to risk of violence and assault, substance misuse and SUD has been shown to also place IPV-EW at an increased risk for future IPV victimization (Kingery et al., 1992; Kilpatrick et al., 1997; Cunningham et al., 2009).

IPV exposure in women may increase the occurrence of risky substance use behaviors. For example, frequent patterns of heavy or binge drinking episodes (Testa and Leonard, 2001; Martino et al., 2005; Weinsheimer et al., 2005; Hink et al., 2015; Ullman and Sigurvinsdottir, 2015) and drinking and driving (Hanson, 2010) have been observed. Elevated rates of illicit substance use (El-Bassel et al., 2005; Gilbert et al., 2012; Hink et al., 2015), misuse of prescription medications (Smith et al., 2012; Hall et al., 2016), and needle sharing for intravenous substance use (Braitstein et al., 2003; Wagner et al., 2009) have also been reported in women exposed to physical or sexual violence relative to non IPV-EW. These risky behaviors contribute to general poor health and increased mortality, even in circumstances where they do not reach criteria for a SUD diagnosis (Patel et al., 2016; Dwyer-Lindgren et al., 2018; Gjersing and Bretteville-Jensen, 2018). For instance, intravenous substance use increases the risk for contracting highly communicable diseases like hepatitis C and human immunodeficiency virus (HIV) as well as other infections (Lake and Kennedy, 2016). In addition, misuse of prescription medications and illicit substances is associated with greater risk of chronic illnesses like pulmonary complications and liver failure (Baltarowich et al., 2018; Nanayakkara and McNamara, 2021).

Diagnostic criteria of SUD are stronger correlates of IPV than consumption patterns alone (Cafferky et al., 2018). IPV-EW are more likely than non IPV-EW to transition from misuse to SUD and have greater SUD symptom severity (Liebschutz et al., 2002; La Flair et al., 2012; Hobkirk et al., 2015). Estimates of 24%–75% (Paone et al., 1992; El-Bassel et al., 2000; Beijer et al., 2018) of women report lifetime IPV exposure at SUD treatment intake. Similarly, the prevalence of SUD in IPV-EW is greater than in the general population (Schneider et al., 2009; Smith et al., 2012; Stone and Rothman, 2019). In one study, half of women presenting to a shelter after IPV exposure report recent alcohol use, almost all of whom met criteria for alcohol use disorder (AUD; Poole et al., 2008). An overrepresentation of IPV-EW in substance use treatment facilities, and vice versa, is one indication that an optimal time to intervene for both IPV and SUD may be when women seek help for either IPV or SUD (Bennett and O’Brien, 2007; Macy and Goodbourn, 2012).

Treatment-seeking samples, however, are not always representative of the population. Self-selection occurs as treatment-seekers are limited to those who are willing and able to attain medical help. Large scale community-based studies may therefore better describe rates of SUD and IPV in the general population. Although the prevalence of co-occurring SUD and IPV are lower in the community than treatment seeking samples, the associative patterns between the two remain strong (Afifi et al., 2009, 2012). Even when controlling for a number of sociodemographic variables and psychiatric comorbidities, women in community samples who report exposure to IPV have a greater occurrence of substance misuse and SUD than those without IPV exposure (Afifi et al., 2012; La Flair et al., 2012; Salom et al., 2015). Interestingly, though, the SUD and IPV associations are often substance-specific and vary by the type of violence exposure, as will be explained in the following sections.

### 2.1. The IPV-SUD temporal relationship

The temporal relationship between IPV and SUD can be difficult to parse as IPV exposure and substance use often occur concurrently (Caetano et al., 2000; Nowotny and Graves, 2013; Simonelli et al., 2014), and retrospective substance use can be difficult to confirm. Most findings describe either cross-sectional or single-time point data, as opposed to time-dependent relationships (Testa and Leonard, 2001; Weinsheimer et al., 2005; Hanson, 2010; Smith et al., 2012; Hink et al., 2015; Ullman and Sigurvinsdottir, 2015; Hohman et al., 2017), although a handful of longitudinal and metanalytic studies have suggested that IPV more often precedes the onset of substance misuse/SUD (Kilpatrick et al., 1997; Testa et al., 2003; El-Bassel et al., 2005; Carbone-López et al., 2006; Devries et al., 2014; Øverup et al., 2015). While some research indicates that current misuse of alcohol (Devries et al., 2014) and other drugs (Kilpatrick et al., 1997; Moore et al., 2008) is associated with future IPV, the current review will focus on substance use patterns that emerge after experiencing IPV (El-Bassel et al., 2005).

### 2.2. Types and patterns of substance use following IPV

Alcohol is arguably the most commonly used psychoactive substance among IPV-EW (El-Bassel et al., 2003; Finney, 2004; Afifi et al., 2012; Kraanen et al., 2014), unsurprising as it is also the most commonly used psychoactive substance in the world (Peacock et al., 2018). Alcohol has acute analgesic properties (Neddenriep et al., 2019; Boissoneault et al., 2020), making it a particularly frequent target of misuse among individuals experiencing physical pain. In-lab alcohol administration paradigms have shown a dose–response relationship between increased blood alcohol content (BAC) and decreased pain intensity, but only once BAC surpasses the legal limit (0.08%; Thompson et al., 2017). This suggests that the use of alcohol as an analgesic requires repeated high doses, a pattern of use that increases the odds of developing AUD (Patrick et al., 2021). Testa et al. (2003) and Øverup et al. (2015) both found a high incidence of heavy/problem drinking episodes in the 1–2 year period following IPV exposure in IPV-EW relative to individuals who did not report exposure to IPV. This was supported by a 2014 meta-analysis that reported a positive association between IPV exposure and subsequent alcohol use in IPV-EW (Devries et al., 2014) although the authors emphasized the need for more multi-wave longitudinal projects due to heterogeneity of measurement and the lack of control for confounds including partner’s alcohol consumption. These studies point to the importance of considering alcohol misuse in the context of pain self-management, particularly in women who may be less likely or able to seek medical treatment for injuries sustained as the result of an IPV-related assault. Reframing alcohol use through this lens may help clinical treatment providers to restructure alcohol-related conversations to reduce stigma and shame.

Among IPV-EW who are able and seek medical care, opiates are prescribed at a staggering rate - up to four times higher than in the general population (Stene et al., 2012; Stone and Rothman, 2019). Although prescription medication use can serve to manage physical pain due to physical abuse (Poole et al., 2008; Cole and Logan, 2010; Hemsing et al., 2016; Stone and Rothman, 2019; Williams et al., 2020), there is a lack of support for long term opiate management of pain, as chronic opiate use decreases pain tolerance (Compton, 1994; Younger et al., 2008) and increases risk of physical dependence (Compton et al., 2003; Raith and Hochhaus, 2004). Further, a prospective medical chart review found that women reporting IPV exposure had higher prescription rates than the general population for opiates and benzodiazepines (Stene et al., 2012), both of which have high addiction potential and can be very dangerous when taken together (Gudin et al., 2013; Afzal and Kiyatkin, 2019). Over-prescription also can drive a transition from prescription opiate to illicit opiate use, like heroin, among treatment-seeking individuals in the general population (Hoffman et al., 2017; Volkow et al., 2019) and non-prescription opiate use has been documented among some IPV-EW (Stone and Rothman, 2019; Williams et al., 2020) and as a consequence of IPV exposure (El-Bassel et al., 2005).

Consistent with alcohol and opiate use patterns, cannabis use disorder is also more prevalent among IPV-EW than in the general population (El-Bassel et al., 2000; Gilbert et al., 2012; Smith et al., 2012). While cannabis has been touted as effective for pain management (Russo et al., 2007; Hill et al., 2017), clinical evidence remains limited, and some argue that cannabis use is more commonly used to cope with psychological as opposed to physical trauma in people exposed to IPV (El-Bassel et al., 2011; Reingle et al., 2012) and a general population of young adults (Bonn-Miller et al., 2008). Cannabis use for stress/coping purposes can increase risk of developing SUD (Hyman and Sinha, 2009) and may be accompanied by a perpetuation of depressive and anxiety disorders and suicidal ideation in adolescents (Copeland et al., 2013). Given the risk for mental health symptoms, such as depression and PTSD, among IPV-EW, and the prevalence of cannabis use in this population, research is needed to explore how cannabis interacts to either exacerbate or ameliorate mental health symptoms (Haney and Evins, 2016; Lowe et al., 2019). Moreover, cannabis use should be considered in context of polysubstance use patterns that can arise from mixing recreational and prescription psychoactive substances. Such multi-drug patterns can exacerbate unsafe and unhealthy behavioral repertoires that can increase risk for revictimization and worsening overall physical and mental health. Thus, although national approval of medicinal and recreational cannabis use is growing, the risk for misuse and dependence in IPV-EW should not be ignored.

IPV-EW have indicated that their substance use can serve as a coping mechanism for the physical, as well as emotional pain of trauma exposure (Øverup et al., 2015; Gezinski et al., 2021). Alcohol and recreational substance use in women is also impacted by partner behavior (Owens et al., 2013; Derrick et al., 2019). In IPV-EW, some perpetrators may coerce or force IPV survivors to use addictive substances (Warsaw and Tinnon, 2018). Added to that is the increased accessibility to addictive prescription medications due to the high likelihood for emergency room visits in this population because of injuries experienced (Kothari and Rhodes, 2006; Rhodes et al., 2011; Hemsing et al., 2016). While reports suggest that a primary driver of substance use in IPV-EW is self-medication, the possibility that both IPV perpetrators and the medical community may exacerbate this behavior warrants significant consideration. Substance users remain highly stigmatized in our society and clinical treatment providers are urged to account for these external factors in the complex needs of IPV-EW.

## 3. Factors affecting the relationship between IPV exposure and substance use

### 3.1. Sociodemographics

Age, education, race, and socioeconomic status are established factors in the risk for IPV exposure (Smith et al., 2018). Racial and ethnic minorities experience disproportionate rates of rape, physical violence, or stalking by an intimate partner (CDC, 2017), and over 80% of Indigenous women in the United States have experienced IPV, stalking, or sexual violence (National Institute of Justice, 2016; Rosay, 2016). Racial discrimination produces increased risk for IPV exposure (Cho et al., 2014) and developing mental health issues after violence exposure (Voth Schrag, 2017). Individuals with disabilities also experience higher rates of IPV (Hughes et al., 2011; Plummer and Findley, 2012; García-Cuéllar et al., 2022) and face additional barriers to help-seeking (Plummer and Findley, 2012). Similarly, racial discrimination increases the risk for developing a SUD (Yoo et al., 2010; Otiniano Verissimo et al., 2014; Pro et al., 2018; Matsuzaka and Knapp, 2020), and racial and ethnic minority groups experience more severe consequences related to SUDs including treatment disparities, criminal justice involvement, morbidity, mortality, and violence (Boyd et al., 2003; Smedley et al., 2003; Iguchi et al., 2005; Amaro et al., 2006; Mennis and Stahler, 2016; Matsuzaka and Knapp, 2020). Moreover, structural bias in the media has promulgated stereotypes about SUDs as a personal deficit specific to people of color, which again influences access to, and the quality of SUD treatment provided to minority groups (Matsuzaka and Knapp, 2020).

Poverty and poor economic conditions are both a risk for and consequence of IPV exposure (CDC, 2019; Fernandes-Alcantara, 2019). Socioeconomic mobility can be restricted through physical and psychological trauma by preventing educational attainment and causing job instability; 64% of IPV-EW report that violence exposure hindered their ability to work (McLean and Bocinski, 2017). Likewise, income inequalities can be perpetuated through substance use related stigma in discriminatory employment and housing practices (Earnshaw, 2020). Gender inequality in education, employment, and income further adds to this burden (Niolon et al., 2017), as does overcrowding, high unemployment rates, neighborhood disadvantage, and low social resource capital (Niolon et al., 2017; CDC, 2019). Moreover, because IPV exposure results in various physical and mental health needs, health care utilization costs are often high and many victims pay for services out of pocket or incur medical debt (McLean and Bocinski, 2017). Economic barriers also negatively impact access to quality SUD treatment (Matsuzaka and Knapp, 2020; CDC, 2022) and often delay treatment seeking due to lack of health insurance and/or reliable transportation (Schmidt et al., 2007; Matsuzaka and Knapp, 2020; CDC, 2022). This delay in treatment seeking can impact the severity of substance misuse issues and the progression to SUD (Lewis et al., 2018; Matsuzaka and Knapp, 2020). Finally, it is noteworthy that adverse childhood experiences (ACEs) including childhood physical and sexual abuse, adversity, and a family history of (or witness to) IPV are associated with increased risk for IPV exposure in adulthood as well as increased risk for substance use (LeTendre and Reed, 2017; CDC, 2018; Currie and Tough, 2021; Leza et al., 2021). For frontline care providers, it is important to consider the intersecting effects of sociodemographics on IPV and substance use risk, particularly as such factors affect the breadth and quality of treatment strategies that are accessible to certain populations. These considerations can inform decisions regarding wraparound care and connection to resources.

### 3.2. Mental health

Exposure to IPV is associated with increased risk for onset of mental health disorders, with incidence rates highest for depression, anxiety, PTSD, and mood disorders (Okuda et al., 2011; Ahmadabadi et al., 2020; Chandan et al., 2020; Mazza et al., 2021). Likewise, SUD demonstrates high comorbidity with mental health conditions and psychiatric disorders (Grant et al., 2015, 2016). Moreover a diagnosis of depression has been associated with subsequent alcohol misuse in IPV-EW (La Flair et al., 2012). Additionally, in IPV-EW recruited from shelter populations and community samples, 60%–90% meet diagnostic criteria for PTSD (Golding, 1999; Dutton et al., 2005, 2006; Woods et al., 2008; Nathanson et al., 2012), and show greater symptom severity than trauma-exposed women who have not experienced IPV (Pico-Alfonso, 2005; Sullivan and Holt, 2008; Woods et al., 2008). In parallel, nearly half of women who develop PTSD following exposure to IPV also develop comorbid SUD (Najavits et al., 2004; Sullivan and Holt, 2008; Sullivan et al., 2012; Najavits and Hien, 2013). Women with a history of physical IPV and comorbid PTSD were nearly 15 times more likely to have days in which they use both alcohol and drugs than IPV-EW without PTSD (Sullivan et al., 2016; McKee and Hilton, 2019). Not surprisingly, the use of alcohol and other drugs to cope with the mental health symptoms following IPV exposure can exacerbate mental health disorders, increase symptom severity, and perpetuate maladaptive coping (Poole et al., 2008; Sullivan et al., 2012; Ullman and Sigurvinsdottir, 2015; Stoicescu et al., 2019). This sets a potentially cyclical relationship between IPV exposure, substance misuse, and mental health problems, which must be considered in all clinical contexts to reduce the potential for worsening mental health outcomes and the risk for revictimization (Gearon and Bellack, 1999; Tol et al., 2019).

### 3.3. Brain injury and substance use

To-date, IPV-related brain injuries have been vastly understudied, yet the rates of partner-inflicted head trauma and probable brain injury among IPV-EW are estimated to be extremely high. For example, studies suggest that anywhere from 30% to 92% of participants report at least one episode of abuse with either exposure to head or neck trauma and/or probable brain injury (Jackson et al., 2002; Valera and Berenbaum, 2003; St Ivany and Schminkey, 2016; Valera and Kucyi, 2017; Esopenko et al., 2021; Campbell et al., 2022). Another recent scoping review found that the prevalence of head trauma is between 19% and 100% across studies, with the large range being due to varied definitions of head trauma and brain injury as well as varied participant inclusion characteristics (e.g., inclusion of only participants with injuries to the head; Haag et al., 2022).

Repetitive exposure to head trauma is also of significant concern as at least 50% of individuals within these samples report multiple episodes of head, neck, and facial trauma (Wilbur et al., 2001; St Ivany and Schminkey, 2016; Valera and Kucyi, 2017; Zieman et al., 2017). Imporantly, each head, neck, and/or facial trauma that occurs as a result of physical and sexual violence in IPV has the potential to result in a brain injury (Capizzi et al., 2020; Esopenko et al., 2021; Meyer et al., 2021; Saleem et al., 2021). IPV-related TBI can occur due to being punched, kicked, thrown, hit with an object, or shaken, all of which can result in focal and/or diffuse axonal injury (Sheridan and Nash, 2007; Valera et al., 2019). NFS, suffocation, and other forms of impeded breathing occurring as a result of IPV can cause hypoxic–ischemic brain injuries from a lack of, or decrease in, oxygen to the brain (Jackson et al., 2002; Valera and Berenbaum, 2003; Kwako et al., 2011; Haag et al., 2022; Valera et al., 2022). With so many avenues for brain trauma, it is probably unsurprising that estimates suggest that approximately 23 million people are living with IPV-related brain injury in the United States alone (St Ivany and Schminkey, 2016).

In the general population, there is some evidence that substance use increases following brain injury, but other factors, such as premorbid substance use, injury severity, and age at time of injury also affect this relationship (Ponsford et al., 2007; Pagulayan et al., 2016; Kennedy et al., 2017; Merkel et al., 2017; Shiwalkar et al., 2017; Schindler et al., 2021). Moreover, the high prevalence of IPV-related head trauma and probable brain injuries, coupled with other factors discussed above (e.g., comorbid mental health symptoms), likely increases the risk of SUD (Oliverio et al., 2020; Oakley et al., 2021). For example, one study of female veterans who all screened positive for TBI found that only the women who also reported a lifetime history of IPV had a SUD diagnosis (Iverson et al., 2020). In the general population, the neural changes and resulting cognitive and psychological impairments occurring after TBI are strong predictors of substance use, misuse, and development of SUD (Graham and Cardon, 2008; Weil et al., 2016; Beaulieu-Bonneau et al., 2018; Olsen and Corrigan, 2021), and contribute to poorer treatment outcomes (Corrigan and Deutschle, 2008; Graham and Cardon, 2008). However, the cause-and-effect relationship between TBI and substance use is complex and hard to parse as substance use is both a risk factor for, and sequelae of, TBI (Nikoo et al., 2017; Eskander et al., 2020). Nonetheless, there is evidence that brain injury often remains undiagnosed among IPV-EW, as substance misuse and mental health issues mask brain injury symptoms, thereby precluding effective assessment and treatment for brain injury (Banks, 2007; Haag et al., 2022). Undiagnosed brain injury has far-reaching mental and physical health consequences, and failure to identify brain injury in the clinical context could prove detrimental to the long-term outcomes of IPV-EW, by impeding delivery of adequate and effective therapies. (Comper et al., 2005). It also can perpetuate stigmas associated with SUD, such as patients being “difficult” and the disease as being intractable.

## 4. Facilitating substance use intervention strategies for IPV-EW

### 4.1. Acknowledging barriers to care

The combined exposure to IPV and SUD creates barriers (i.e., women with SUDs being denied shelter admission) that inhibit access to treatment for either IPV or SUD (Logan and Walker, 2004; Humphreys et al., 2005; Klostermann, 2006; Macy and Goodbourn, 2012). Standard trauma-informed frameworks are not always sufficient to treat the complex needs of IPV survivors who are also coping with substance use problems (Macy and Goodbourn, 2012; Capezza et al., 2015), and SUD treatment facilities rarely screen for IPV exposure (Bennett and Lawson, 1994; Collins et al., 1997; Logan et al., 2002). Another consideration is encouragement or coercion by partners to continue drug use (Gilbert et al., 2001; Simonelli et al., 2014; USDHHS, 2020). For instance, partner opposition to SUD treatment entry poses a barrier to recovery (Amaro and Hardy-Fanta, 1995; USDHHS, 2020), and IPV perpetrators may keep substances around the home and pressure or force their partner to use substances (USDHHS, 2020). Additionally, brain injury-related cognitive and neurobehavioral impairments can make it harder for those with brain injury and IPV exposure to gain access to, stick with, and benefit from standard treatments for SUD (Vungkhanching et al., 2007; Olson-Madden et al., 2012). These barriers , in general SUD samples, have been shown to undermine the likelihood of SUD treatment success (Bates et al., 2006, 2013). Thus, the creation and implementation of treatment options that include cognitive and neurobehavioral support remain urgently needed.

### 4.2. Screening

Given that mental health diagnoses, including SUD, increase the risk of continued and future victimization (Gearon and Bellack, 1999; Friedman and Loue, 2007), it would be beneficial for all emergency departments and mental health facilities to screen for history of IPV, and associated comorbidities, particularly exposure to head, neck, and facial trauma and probable brain injury, in anyone seeking treatment for these conditions (Waalen et al., 2000; Clark et al., 2008; Rabin et al., 2009; Radcliffe and Gilchrist, 2016; Gilchrist and Hegarty, 2017; Iverson et al., 2020). Likewise, screening for IPV and brain injury in substance use treatment programs is also needed as efficacy is significantly reduced in IPV-EW who are not receiving care and resources related to violence exposure (Capezza et al., 2015). Similarly, screening for SUD in anyone seeking IPV treatment and shelter services not only increases the safety of IPV populations, as research shows SUD is linked with re-victimization, but will also provide timely SUD treatment (Kilpatrick et al., 1997; Capaldi et al., 2012; Simmons et al., 2014, 2017; McKee and Hilton, 2019). Free, validated self-report tools, such as the Alcohol Use Disorders Identification Test (AUDIT; Saunders et al., 1993), the Drug Abuse Screening Test (DAST-10; Skinner, 1982; Bohn et al., 1991), and the Alcohol, Smoking, and Substance Use Involvement Screening Test (ASSIST; Humeniuk et al., 2010) are readily available and can be used to quickly assess alcohol and substance misuse. Health care agencies could also serve a greater number of IPV-EW who need SUD treatment, but who may lack health insurance coverage and/or are financially dependent on an IPV perpetrator (Clark et al., 2008; Foster et al., 2010; Priester et al., 2016), by including an assessment of financial need to ensure access to services regardless of financial limitations (Hageman and George, 2018). Screening processes may best be facilitated by social work staff trained in resource facilitation and could increase the likelihood that basic needs are also met (Macy et al., 2013). Lastly, key stakeholders, including domestic violence and SUD agencies could further improve outcomes by screening and referring IPV-EW for brain injury-related services, as cognitive deficits resulting from brain injury can make a survivor’s physical and emotional healing more difficult (Iverson and Pogoda, 2015; Haag et al., 2022) and, more generally, SUD treatment less efficacious.

### 4.3. CBT-based interventions

Intervention research shows support for cognitive-behavioral therapies (CBT) as most effective for targeting PTSD, depression, emotional well-being, and substance misuse in IPV groups (Eckhardt et al., 2013; Arroyo et al., 2017). A systematic review and meta-analysis found that CBT-based interventions specifically tailored for the unique needs of IPV survivors resulted in the largest effect sizes for outcomes such as PTSD, depression, and self-esteem with moderate effect size decreases in substance use outcomes (Arroyo et al., 2017). Other recent research demonstrates the efficacy of CBT with interventions such as Helping to Overcome PTSD through Empowerment [HOPE] (Johnson and Zlotnick, 2009). This CBT-based HOPE intervention was developed specifically for IPV-EW who were also residing in a shelter and was shown to reduce the likelihood for revictimization. Cognitive Trauma Therapy for Battered Women (CTT-BW) is another intervention developed to address trauma history, exposure to abuse and abuser reminders, as well as monitoring of negative self-talk and cognitive therapy for guilt (Kubany et al., 2004). CTT-BW showed significant reductions in PTSD and depressive symptoms among IPV-EW that were maintained at 6-month follow-up (Kubany et al., 2004).

### 4.4. Concurrent IPV/SUD treatment

There is evidence that concurrent IPV services and substance use treatment may be a more effective approach than treating IPV or SUD on their own (Capezza and Najavits, 2012; Macy and Goodbourn, 2012; Capezza et al., 2015). For example, one treatment strategy designed to address both trauma symptoms and SUD, known as Seeking Safety, has been efficacious in reducing SUD and PTSD symptoms (Najavits, 2007) and has been recommended for use with IPV groups (Cohen et al., 2013; McKee and Hilton, 2019). By utilizing one facility with the same team of treatment personnel, coordination is efficient, cost-effective, and there is a greater likelihood that individuals will attend, complete, and benefit from the program (Mueser et al., 2003; Poole et al., 2008; Murphy and Ting, 2010; Capezza et al., 2015; McKee and Hilton, 2019).

### 4.5. Teaching and strengthening resilience

A crucial consideration for substance use treatment programs for IPV-EW is resilience, a process of positive adaptation despite adverse conditions (Crann and Barata, 2016; Tsirigotis and Łuczak, 2018; Rollero and Speranza, 2020; Gonzalez-Mendez and Hamby, 2021). Resilience is a skill set that promotes beneficial responses to avoid negative outcomes and reduce harmful effects on physical and psychological functioning (Luthar et al., 2000; Humphreys, 2003; Gonzalez-Mendez and Hamby, 2021). Among IPV-EW, higher resilience has been consistently correlated with lower levels of PTSD, depression, anxiety, psychological distress, and risk for substance use (Humphreys, 2003; Anderson et al., 2012; Gonçalves and Matos, 2020). Interventions that can both teach and strengthen resilience have been shown to improve IPV-EW’s confidence, independence, power, and positive social relationships, all of which contribute positive outcomes (Humphreys, 2003; Decker et al., 2020). Research has shown that IPV-EW who employ strategies such as physical activity, creativity, spirituality, introspection, and optimism are more likely to demonstrate greater resilience, positive adaptation, self-efficacy, and healing from abuse (Drumm et al., 2014; López-Fuentes and Calvete, 2015). Similarly, interventions that empower IPV-EW to access and use their strengths (e.g., social resources, help-seeking behaviors, assertiveness, problem-solving skills) enable survivors to respond to partner violence and related sequelae with healthier behavioral strategies, ultimately resulting in a decreased risk for substance use problems (Luthar et al., 2000; Humphreys, 2003; Sani and Pereira, 2020).

Several studies have documented that an increase in resilience is associated with decreased substance use and SUD recovery (Elm et al., 2016; McKinley and Theall, 2021; Yamashita et al., 2021). In fact, one study demonstrated that women with greater resilience, defined as a strong sense of self, an awareness of the abuse, a knowledge of resources, and a future hope, showed more awareness of how their abuser’s and their own substance use contributed to the maintenance of the abusive relationship (Werner-Wilson et al., 2000). This increased awareness is a crucial factor needed for ending the potentially cyclical nature of substance use in IPV (Gutierres and Van Puymbroeck, 2006). In addition, increased resilience is associated with healthier and safer decision making in IPV-EW which supports efforts to reduce risk for revictimization (McFarlane et al., 1997; Humphreys, 2003; Decker et al., 2020; Schaefer et al., 2021). The ability of resilience to reduce physical and psychological distress, while improving overall health, could make it a key target for recovery from SUD (Gorvine et al., 2021). Teaching and strengthening resilience could provide a positive, empowering, and healthy alternative for IPV-EW to obtain relief from the physical pain and psychological trauma symptoms that may be underlying the use of alcohol and other drugs (Hernandez and Mendoza, 2011; Gorvine et al., 2021). With prior research demonstrating the power of resilience to reduce substance use and misuse, future research is necessary to expand the potential for SUD prevention in IPV using the resilience framework (Gorvine et al., 2021; Yamashita et al., 2021).

## 5. Conclusion

The complex and cyclical relationship between IPV exposure and substance misuse warrants significantly more research and clinical attention, particularly considering that both demonstrate complex interactions with with highly comorbid factors, such as mental health problems and brain injury. Critical next steps include cross-fertilization of ideas, theories, and data from fields such as social work, psychology, neuroscience, addiction, and women’s health. This review presents evidence for an elevated risk for misuse of recreational and prescription substances in this population, but emphasizes a need for clinicians to reframe perspectives on this use to improve outcomes for all IPV-EW. Clinicians are urged to consider substance use in light of physical and emotional pain that often results from violence exposure, and take care to screen for SUDs prior to prescribing potentially addictive medications. Mental health disorders and IPV-related brain injuries are additional factors for clinicians to consider. It is only when substance use, mental health, and brain health are considered together that IPV-EW will gain access to optimal treatment strategies. In the absence of such comprehensive screening, IPV-EW may appear unable or unwilling to affect meaningful change to improve their quality of life, thereby perpetuating the stigma and bias that remains deeply entrenched against IPV survivors and substance users.

The suggested clinical approach, derived from the evidence reviewed, is for concurrent treatment of IPV and SUD, with added infrastructure for mental health and cognitive support to facilitate treatment seeking, treatment gains, and long-term quality of life - both in terms of violence exposure and substance use. Clinicians treating those with IPV should consider SUD, mental health diagnoses, and cognitive impairments in terms of clinical presentation and severity. Likewise, those providing SUD treatment should assess for IPV exposure as well as mental and brain health, as this may strongly alter the optimal treatment path (WHO, 2013). It is acknowledged, however, that such comprehensive screenings are time and resource intensive, and may not be feasible in all contexts. Yet, even for those with limited time and resources, such as emergency department health care providers and shelter workers, building knowledge of these comorbidities is essential for improving outcomes for IPV-EW. Future research should be directed at probing the interplay of SUD, brain injury, mental health, and IPV, as well as testing which screening and recovery protocols provide the best treatment outcomes in IPV-EW experiencing SUD across different contexts.

## Author contributions

CE, JB, EW, DT, AM, and KD-O’C were involved in manuscript conceptualization. JM, EB, CE, and JB wrote the initial paper draft. JP and NS provided editorial and conceptual feedback. All authors contributed to the article and approved the submitted version.

## Funding

CE, EW, and DT are supported by the National Institutes of Neurological Disorders and Stroke [R01NS115957]. DT is also supported by the and the National Institute of Neurological Disorders and Stroke [R01NS122184; R61NS120249]. JB is supported by a NIH/NIAAA K-award [K02AA025123]. JP is supported through the NIH postdoctoral fellowship training program [T32AA028254]. KD-O’C is supported by the National Institute on Disability, Independent Living, and Rehabilitation [90DPTB0009] and the International Alzheimer’s and Related Dementias Research Portfolio [1RF1NS115268].

## Conflict of interest

The authors declare that the research was conducted in the absence of any commercial or financial relationships that could be construed as a potential conflict of interest.

## Publisher’s note

All claims expressed in this article are solely those of the authors and do not necessarily represent those of their affiliated organizations, or those of the publisher, the editors and the reviewers. Any product that may be evaluated in this article, or claim that may be made by its manufacturer, is not guaranteed or endorsed by the publisher.

## Footnotes

^1^Brain injury will be used as an umbrella term for IPV-related TBI and/or hypoxic–ischemic brain injury (described in section 3.3).
